# Upregulation of TCF21 inhibits migration of adrenocortical carcinoma cells

**DOI:** 10.1007/s12672-021-00417-6

**Published:** 2021-07-23

**Authors:** Jean Lucas Kremer, Thais Barabba Auricino, Bárbara dos Santos Passaia, Claudimara Ferini Pacicco Lotfi

**Affiliations:** Department of Anatomy, Institute of Biomedical Sciences, University of São Paulo, São Paulo, Brazil

**Keywords:** Adrenocortical carcinoma cells, TCF21, Methylation, Migration, Matrix metalloproteinases

## Abstract

**Background:**

Adrenocortical carcinomas (ACC) are rare and aggressive cancer. Our previous study has revealed that the transcription factor 21, TCF21, is downregulated in ACC and regulates steroidogenic factor 1 (SF-1) binding to the *SF-1* E-box promoter. In addition, it could be found that TCF21 is a predictor of overall survival (OS) in adult carcinomas.

**Methods:**

In this study, it was investigated the correlation between *TCF21* expression and the promoter methylation status in adrenocortical tumor cells, carcinomas and adenoma. The biological function and potential molecular mechanism of TCF21 restoration in migration and invasion of ACC cells was examined.

**Results:**

We could be demonstrated a negative correlation between the level of *TCF21* expression and methylation of its promoter in adenoma and carcinoma cells indicating the epigenetic control of *TCF21* expression. It was also demonstrated that the expression of *TCF21* inhibits migration and invasion in the ACC cell line, H295R cells, using plasmid transfection to express *TCF21*. Furthermore, it could be investigated the TCF21 function as tumor suppressor probably through Kisspeptin 1 (KISS-1) expression and epithelial–mesenchymal transition (EMT) reversion, as well as the modulation of several metalloproteinases in ACC cells.

**Conclusions:**

Our results suggest that enhancement of *TCF21* expression levels may be a potential strategy to revert invasive abilities in adrenocortical carcinomas.

**Supplementary Information:**

The online version contains supplementary material available at 10.1007/s12672-021-00417-6.

## Introduction

Adrenocortical tumors are usually incidentally diagnosed in 6–7% of the population [1]. Most of them are benign and non-functioning classified as adenomas (ACA), requiring only clinical monitoring [2]. In contrast, adrenocortical carcinomas (ACC) are rare with incidence of 1–2 cases per million and are aggressive and metastatic tumors [3]. Transcription factor 21 (TCF21) also known as capsulin, epicardin, and Pod1 belongs to the basic helix–loop–helix (bHLH) family and locates at chromosome 6q23-q24 [4–6]. The TCF21 encodes a transcription factor which binds DNA as a heterodimer through the E-box CANNTG sequence. The normal function of TCF21 is to promote the mesenchymal–epithelial transition (MET) during the development and differentiation process [7, 8]. The downregulation of TCF21 reverts the MET process favoring migration and tumor invasion as reported in colorectal tumors, esophageal squamous cell carcinoma and urogenital cancers [9–11]. Thus, it could be reported that TCF21 is downregulated in ACC and regulates the steroidogenic factor 1 (SF-1) and StAR protein (steroidogenic acute regulatory protein) binding to the *SF-1* E-box promoter in adrenocortical tumor and normal adrenal cells [12, 13]. In addition, it could be found that TCF21 is a predictor of overall survival (OS) in adult carcinomas [14]. The epigenetically inactivation of *TCF21* is associated with regulation of epithelial–mesenchymal transition (EMT), invasion, metastasis, cell cycle, and autophagy in different tumors, and might be an important role in tumor development [15, 16]. However, no studies have reported on the role of TCF21 in migration and invasion of ACC, and the molecular mechanism is unknown. One of common mechanism in many human tumors is the hypermethylation-mediated silenced expression of *TCF21*, which has not yet been studied in adrenocortical tumors [17, 18]. In the present study, it was investigated the correlation between TCF21 expression and the promoter methylation status in adrenocortical tumor cells, carcinoma and adenoma cells, and the biological function and potential molecular mechanism of TCF21 in motility of ACC cells. Our results demonstrated that there is a negative correlation between the level of *TCF21* expression and methylation of its promoter. Upregulation of *TCF21 *in vitro inhibited migration and invasion functions expressively, which could act partly through involvement of EMT reversion and Kisspeptin 1 (KISS-1) and metalloproteinases (MMPs) expression.

## Materials and methods

### Tumor cell cultures

Human adrenocortical carcinoma cell line NCI-H295R [19] obtained from American Type Culture Collection (ATCC) was used. H295R were cultured in Gibco RPMI medium with 2% Fetal Bovine Serum (FBS) and 1% Insulin Transferrin-Selenium (ITS). Secondary cell culture ACC-T36 was obtained from adult patient diagnosed with ACC as described in [12]. ACAPed-T7 pediatric secondary cell culture were obtained from a functioning adrenocortical adenoma as described in Almeida et al. [20]. Both secondary cell culture was maintained in Dulbecco’s Modified Eagle Medium (DMEM) with 10% FBS and used until the fourth or sixth passage. All cultures were maintained at 37 °C in a 95% air 5% CO_2_ and humidified environment. All cell cultures were regularly authenticated by STR DNA profiling analysis, and mycoplasma contamination was excluded by using qPCR Mycoplasma test kit (PanReac AppliChem, ITW Reagents, Illinois, Chicago, USA).

### Methylation analysis of TCF21 promoter

The bisulfite conversion was performed using EZ DNA Methylation Gold kit (Zymo Research Cat No. D5005), according to the manufacturer’s protocol. As a control, a Human Methylated & Non-methylated DNA Set kit (D5014-1-Zymo Research) following the Polymerase Chain Reaction (PCR) conditions was used, according to the manufacturer. A regulatory region of the *TCF21* promoter was selected [21, 22] and a sequence of 600 bp just before the start of the transcription site (TSS) was used as showed in Table 1S. DNA samples were amplified by conventional PCR using the Hot-Start Platinum DNA Polymerases enzyme (Thermo Fisher Scientific) according to the manufacturer’s protocol, using the primers 5ʹ-TCACCATAAAGATTCTAGGAAGCA-3ʹ and 5ʹ-GAGCGAGCGGCTGAGGAAT-3ʹ for control DNA, and 5ʹ-TTATTATAAAGATTTTAGGAAGT-3ʹ and 5ʹ-GAGYGAGYGTTGAGGAAT-3ʹ for bisulfite-converted DNA. Primers were selected using the tool “bisulfite primer seeker” from Zymo Research^®^ (https://www.zymoresearch.eu/bisulfite-primer-seeker). PCR reaction was analyzed on an agarose gel (1%) by electrophoresis and the product was purified using Exonuclease 1 (Applied Biosystems) and Shrimp Alkaline Phosphatase—SAP (Applied Biosystems), according to the manufacturer. For Sanger sequencing, the primers 5ʹ-TCACCATAAAGATTCTAGGAAGCA-3ʹ for control DNA, and 5ʹ-TTATTATAAAGATTTTAGGAAGTA-3ʹ for bisulfite-converted DNA were used. The reaction was prepared using the BigDye^®^ Terminator v3.1 Cycle Sequencing Kit, performed in the ABI 3730 DNA Analyzer (Life Technologies, Applied Biosystems). The data were analyzed by DNA Sequence Assembler^®^ software comparing sequenced bases without and with the bisulfite-conversion. The control was performed using the database with the sequence of the TCF21 gene (ENSG00000118526; *r* = 6: 133,889,138–133,895,553) GeneCards^®^ The Human Gene Database.

### Demethylation by 5-aza-2ʹ-deoxycytidine

3.0 × 10^5^ cells were seeded in triplicate in a 12-well multiwell plate. After 24 h, the cells were treated with different concentration (75, 100, 150, 200 µM or not treated) of 5-aza-2ʹ-deoxycytidine (Sigma-Aldrich USA) for 24 h; or treated with 100 µM for 12, 24, 48, 72 h or not treated. The qRT-PCR was performed as described below and the experiments were performed three times.

### qRT-PCR

2.0–8.0 × 10^5^ cells were seeded and after 48 h total RNA was extracted using Trizol^®^ (Invitrogen) as described by the manufacturer. The RNA integrity and its concentration were evaluated by agarose gel electrophoresis (2%) and spectrometry (NanoDrop 2000c, Thermo Fisher Scientific, Waltham, MA, USA). The cDNA was generated from 1 µg of RNA using M-MLV Reverse Transcriptase (Invitrogen, USA). The qRT-PCR was performed using the 7500 Real Time PCR System Sequencer (Applied Biosystems, Foster City, CA, USA) and Sybr Green reagents (Applied Biosystems) and primers (Table 1). A cycle threshold (CT) value was selected in the linear range of amplification for each sample in triplicate and was normalized by endogenous control genes GUSB (human β-glucuronidase) or β-actin. The relative expression levels were calculated using the 2^−ΔΔCt^ method [23], where ΔΔCt is the difference between ΔCt value of a given sample and the ΔCt of commercial normal human adrenal pool (NA) (BioChain, USA). The data from three different experiments were presented as the mean ± SD.

**Table 1:** Sequence of primers used in the qRT-PCR assay

Gene	Forward	Reverse
*β actin*	TGGTGATGGAGGAGGTTTAGTAAGT	AACCAATAAAACCTACTCCTCCCTTAA
*GUSB*	GACACGCTAGAGCATGAGGG	TCTGCATAGGGGTAGTGGCT
*TCF21*	GAAAGAAGTGGTGACCGCGA	GTAAAGTGTTCTCGCGGGGT
*MMP8*	AACGCACTAACTTGACCTACAG	CTCCAGAGTTCAAAGGCATCC
*MMP9*	ACGCACGACGTCTTCCAGTA	CCACCTGGTTCAACTCACTCC
*MMP2*	CTCATCGCAGATGCCTGGAA	TTCAGGTAATAGGCACCCTTGAAGA
*MMP14*	GCCTTCTGTTCCTGATAA	CCATCCTTCCTCTCGTAG
*VIM*	GCAAAGATTCCACTTTGCGT	GAAATTGCAGGAGGAGATGC
*KISS-1*	ACCTGGCTCTTCTCACCAAG	TAGCAGCTGGCTTCCTCTC
*TIMP-1*	TTGTGGGACCTGTGGAAGTA	CTGTTGTTGCTGTGGCTGAT

*GUSB* Human β-glucuronidase, *TCF21* Transcription factor 1, *MMP-8* metalloproteinase 8, *MMM-9* metalloproteinase 9, *MMM-2* metalloproteinase 2, *MMM-14* metalloproteinase 14, *VIM* Vimentin, *KISS-1* Kisspeptin 1, *TIMP-1* metalloproteinase inhibitor 1 precursor

### Transfection assays

1.0 × 10^6^ NCI-H295R cells were seeded and transiently transfected with 4 µg with pCMVMycPod1 [24] or the empty vector pCMVMyc and 8 µl of Turbofect (Thermo Fisher Scientific, Waltham, MA, USA) for 5 h. To transient transfection with pCMV-MMP-8 or pcDNA-MMP-9, 5 × 10^6^ cells were seeded and transfected with 8 or 12 µg of plasmid DNA and 12 µl of Turbofect, for 24 h. After 24 h of transfection, total RNA was extracted with Trizol (Invitrogen). Three independent total RNA extractions were performed. 1.5 × 10^5^ ACAPed-T7 cells were seeded and 24 h later transfected with a mixture of siRNAPOD1-HSS144226 and siRNAPOD1-HSS144228 (Thermo Fisher Scientific) or with RNAi Human Actin positive/negative control^®^ (Cat. Numb. 12935141, Thermo Fisher Scientific) to a final concentration of 100 nM and 9 µl of RNAiMax Lipofectamine (Invitrogen, Carlsbad, CA, USA) for 72 h.

### Immunoblotting

3.0–8.0 × 10^5^ were seed and 48 h later lysed in RIPA buffer containing protease and phosphatase inhibitors (Sigma Aldrich, Germany). The total protein concentration was determined using the Bradford assay. 20 µg of total protein was resolved in 12% polyacrylamide gel, transferred to a nitrocellulose membrane and staining with Ponceau. Non-specific binding sites were blocked with 5% Bovine Serum Albumin (BSA) or 5% non-fat dried milk in TRIS-buffered saline solution containing 1% Tween 20 (TBST). The primary antibodies utilized were anti-MMP-9 (Santa Cruz, monoclonal antibody sc-21733, 1:1000) and anti-βactin (Santa Cruz, sc-47778, 1:2000) in TRIS-buffered saline containing 1% Tween 20. Proteins were visualized using Enhanced Chemiluminescence (ECL) (Amersham Hybond ECL, Freiburg, Germany) detection with secondary HRP-conjugated anti-rabbit (cod. 711-035-152, Jackson ImmunoResearch Inc., West Grove, PA, USA, 1:4000) or anti-mouse (cod. 515-035-062, Jackson ImmunoResearch Inc., West Grove, PA, USA, 1:4000) antibodies. Image J software was used to quantified immunoblotting results. The data from three different experiments were presented as the mean ± SD.

### Migration and invasion analysis

5.0 x 10^5^ cells were seeded in NeuroProbe^®^ 10-well chemotaxis chamber (8 µm pores, Cat. Nº AA10) containing medium with 0.1% FBS and chemoattracted with complete culture medium for 24 h. Invasion assay was performed in 6 well Transwell^®^ permeable inserts (8 µm pores) (Corning, Cat. Nº 3422), 50 µl/cm^2^ of Matrigel^®^ (5 mg/ml, Corning, Cat. Nº 354248) and chemoattracted for 48 h. The membrane or inserts was fixed in 4% paraformaldehyde and stained with Giemsa. Five fields per membrane were analyzed under an optical light microscope (100× magnification) using the NIS-Element Nikon image analysis program.

### Statistical analysis

Data were analyzed using unpaired *t* test or Analysis of Variance (ANOVA), when indicated, by GraphPad Prism 8 software. The results were considered statistically significant when *p* < 0.05.

## Results

### Negative correlation between the level of expression of TCF21 and methylation of its promoter

**Fig. 1:**
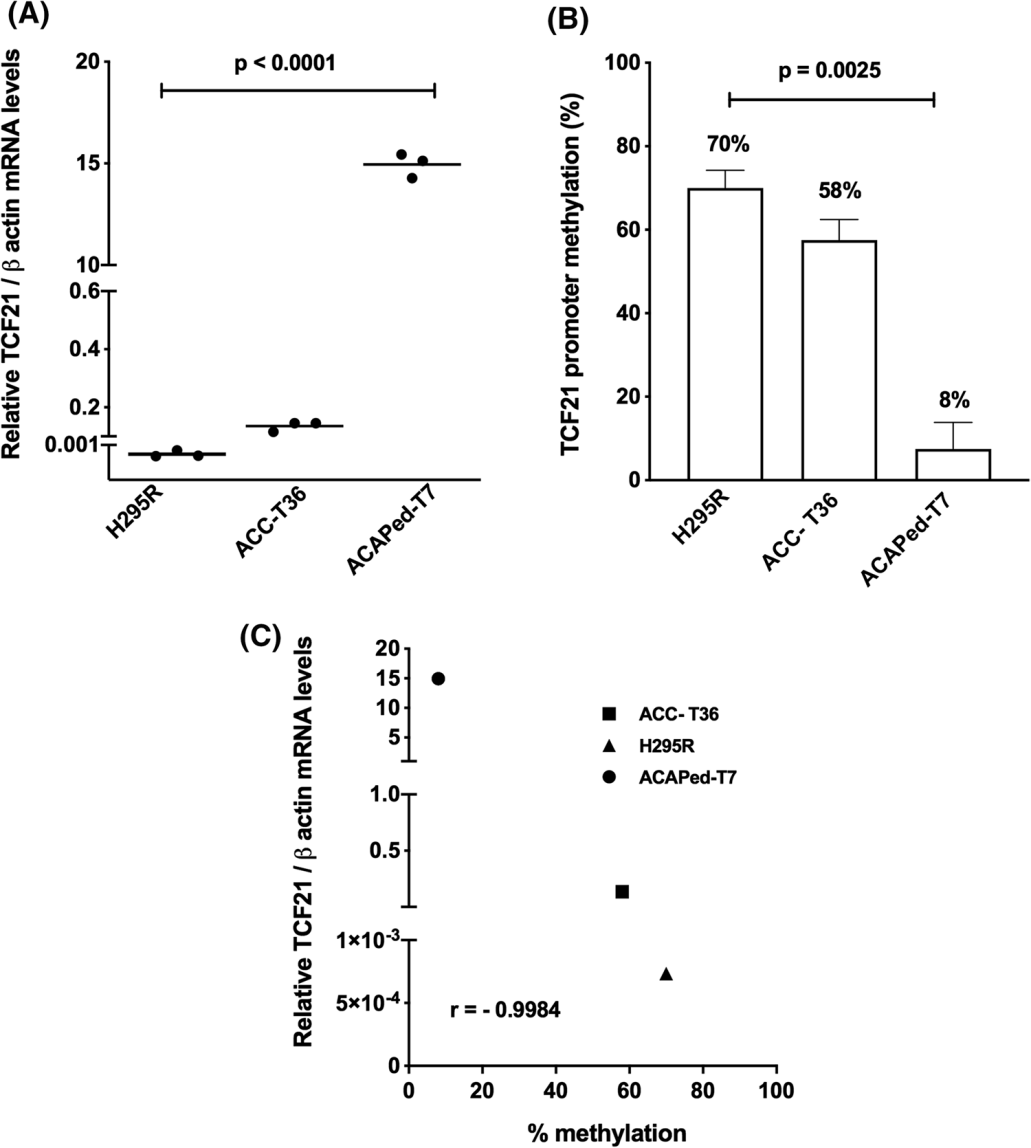
TCF21 expression and promotor methylation. **A** Relative *TCF21* expression in adrenocortical tumor cells; **B** Methylation levels of *TCF21* promoter; **C** Negative Pearson’s correlation between *TCF21* expression and level of promoter methylation (*r* = − 0.9984). NA normal adrenal pool; ACC-T36 adrenocortical carcinoma cell culture from patient; ACAPed-T7, pediatric adrenocortical adenoma cell culture from patient; H295R cell line. The experiments were performed in triplicate and repeated three times. The results were expressed as the mean ± SD. Statistical significance was assessed by One-way ANOVA

Analysis of *TCF21* expression in different adrenocortical tumor cell cultures (Fig. 1A) showed lower mRNA expression of *TCF21* in human ACC cells; H295R cells and ACC-T36 cells when compared to cell culture from pediatric adenoma, ACAPed-T7 and human normal adrenal pool (NA), which was used as reference. These results of *TCF21* expression level in vitro are in agreement with previous study in a cohort of adrenocortical tumor samples from patients [12, 14, 25]. Sequencing for analysis of methylation of the *TCF21* promoter in ACC cell cultures, H295R and ACC-T36 cells, showed 70% and 58% methylation of *TCF21* promoter, respectively, whereas in ACAPed-T7 was found 8% methylation (Fig. 1B). The Pearson’s correlation coefficient of *TCF21* promoter methylation in different cell types was negative (*r* = − 0.9984) in agreement with the level of mRNA TCF21 expression (Fig. 1C). To confirm the regulation of *TCF21* by promoter methylation, H295R cells were treated with demethylating agent 5-aza-2-deoxycytidine (5-aza). 100 µM of 5-aza for 48 h increased the relative expression of *TCF21* mRNA level reversing the hypermethylation condition of the TCF21 promoter (Figure 1S).

### Modification of *TCF21* expression level in adrenocortical tumor cells

**Fig. 2:**
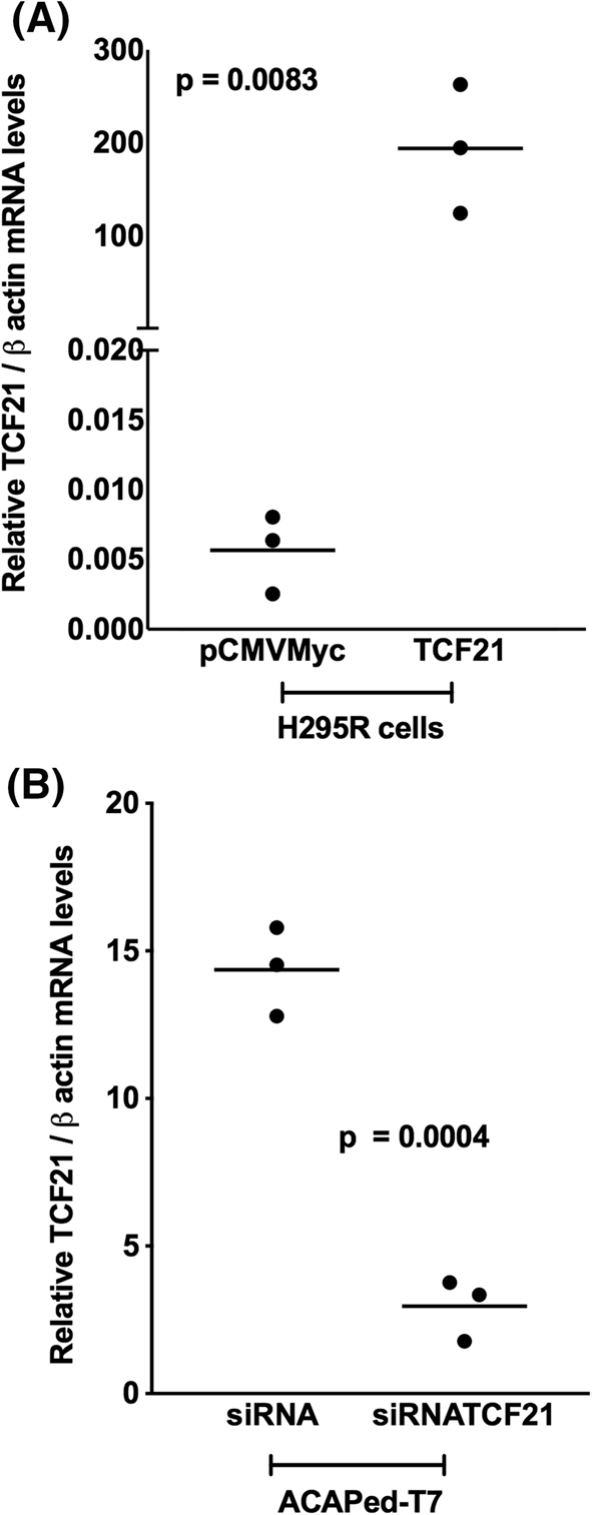
Modulation of TCF21 expression. **A** *TCF21* overexpression in H295R transfected with pCMVMycPod1 or empty vector (pCMVMyc); **B** Inhibition of *TCF21* in ACAPed-T7 cells using siRNATCF21 or control (siRNA). The experiments were performed in triplicate and repeated three times. The results were expressed as the mean ± SD. Statistical significance was assessed by unpaired *t* test

To explore the biological function of TCF21 in tumor adrenocortical cells, we utilized pCMVMyc-Pod1 transfection to increase *TCF21* expression levels in H295R cell line (Fig. 2). In H295R cells, *TCF21* was overexpressed (194.1 ± 39.9; *p* = 0.0083) compared to control (empty plasmid) (Fig. 2A). To inhibit the *TCF21* expression, we used siRNA in ACAPed-T7 cells that expressed *TCF21* constitutively (Fig. 1A). *TCF21* was significantly lower (− 11.4 ± 1.1; *p* = 0.0004) in ACAPed-T7siRNATCF21 compared to control, ACAPed-T7siRNA cells (Fig. 2B).

### Upregulation of *TCF21* reduces ACC cell migration and invasion

**Fig. 3:**
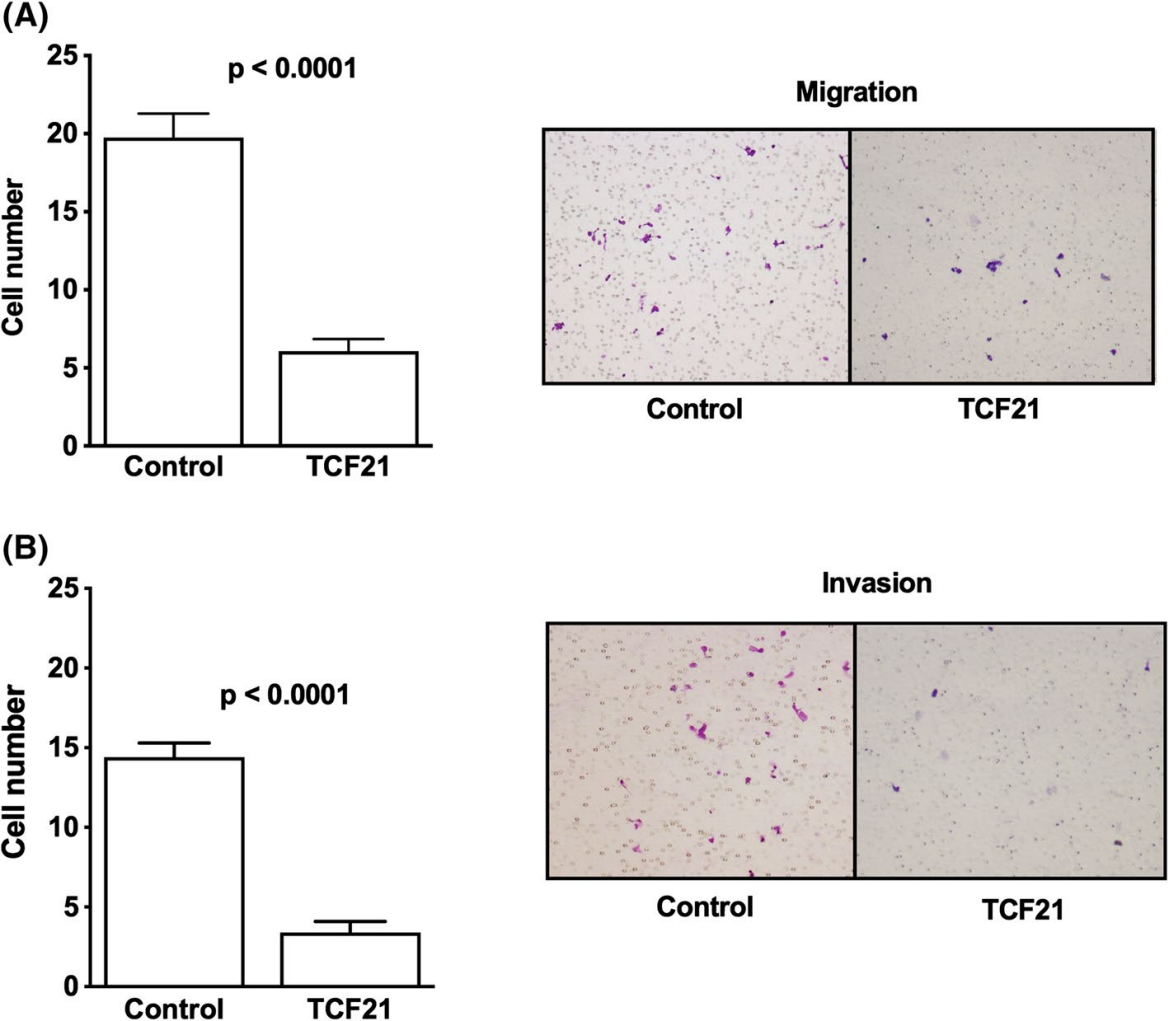
Upregulation of TCF21 decrease ACC cell migration and invasion. **A** Migration of H295RpCMVMycPod1 cells (TCF21) was inhibited in relation to respective controls and representative stained membranes; **B** Invasion of H295pCMVMycPod1 cells (TCF21) was inhibited in relation to respective controls and representative stained membranes. The experiments were performed in triplicate and repeated three times. Magnification of membranes (100×). The results were expressed as the mean ± SD. Statistical significance was assessed by unpaired *t* test

To examine the effect of TCF21 on migration and invasion, Transwell assays were performed using H295R/TCF21 and the corresponding control cells (Fig. 3). The results indicated that the number of migrated and invaded cells was lower in H295R/TCF21 than control cells, indicating that the increase of *TCF21* has a negative effect on the migration and invasion of ACC cells in vitro.

### Inhibition of TCF21 expression promotes ACA cell migration and invasion

**Fig. 4:**
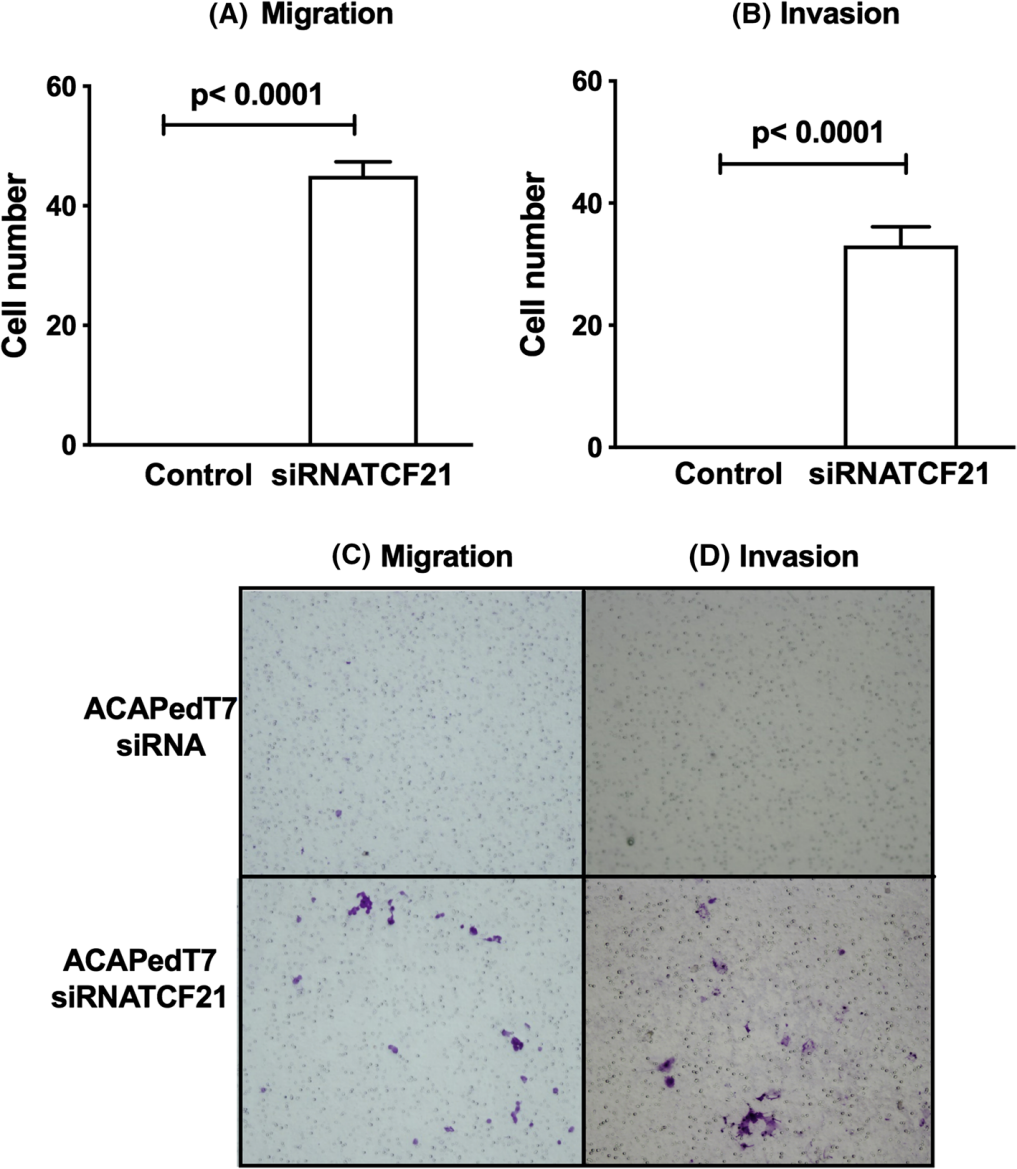
Downregulation of TCF21 promote ACA migration and invasion. **A**, **C** Migration of ACAPed-T7siRNATCF21 cells in relation to control (ACAPed-T7siRNA); **B**, **D** Invasion of ACAPed-T7siRNATCF21 cells in relation to control (ACAPed-T7siRNA). The experiments were performed in triplicate and repeated three times. The results were expressed as the mean ± SD. Statistical significance was assessed by unpaired *t* test

To confirm the involvement of TCF21 in adrenocortical tumor mobility, *TCF21* expression was efficiently inhibit in ACAPed-T7 cells by siRNATCF21 as showed in Fig. 2B. Migration and invasion assays using ACAPed-T7siRNATCF21, and respective control cells, showed that inhibition of *TCF21* enable the migration and invasion of adrenocortical cells when compared to control cells (Fig. 4). Taken together, these data indicate that adrenocortical tumor cells become less likely to migrate upon presence of TCF21 expression.

### TCF21 increased the expression of anti-invasive effectors and downregulate pro-invasive effectors in ACC cells

**Fig. 5:**
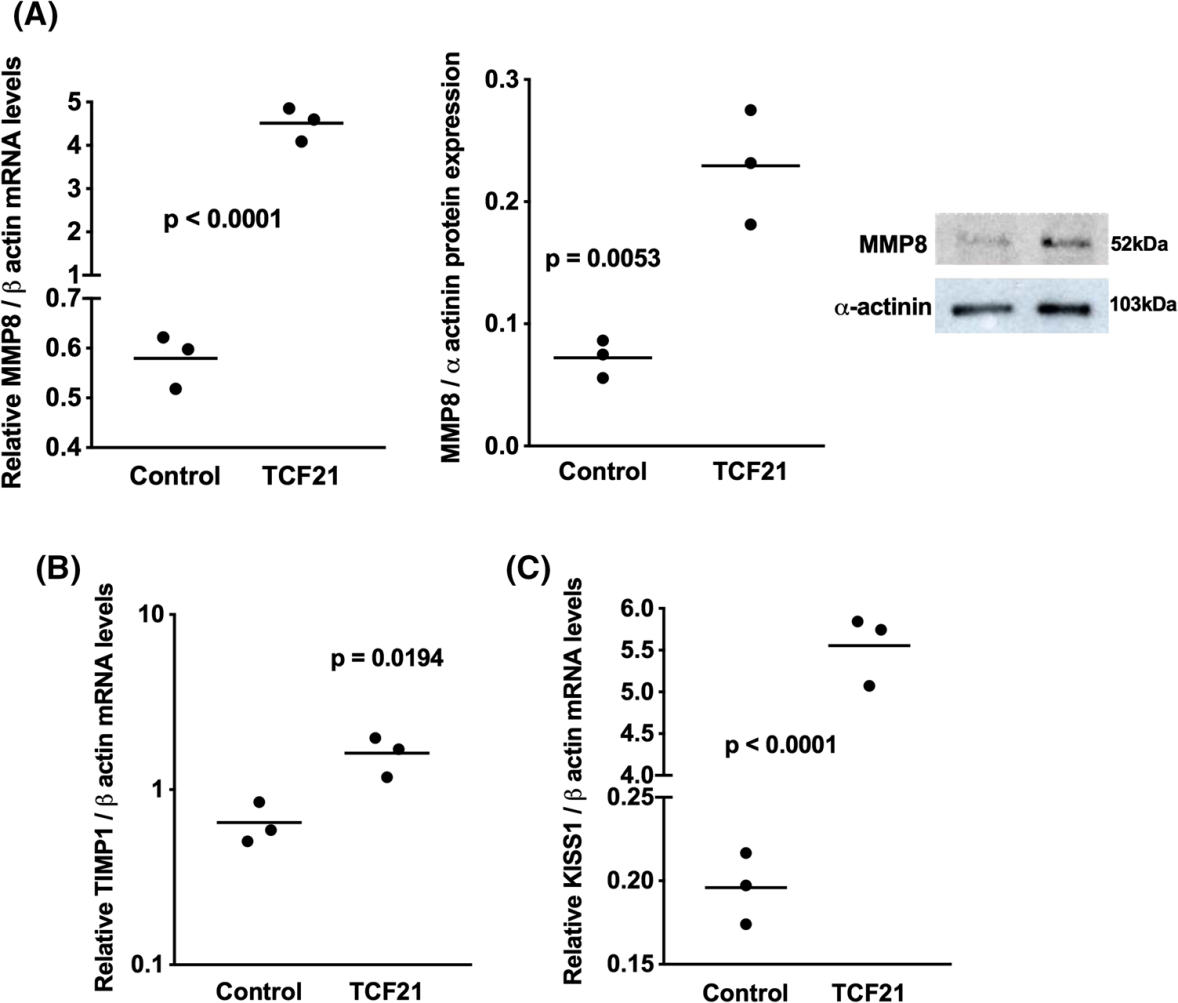
TCF21 increased the expression of anti-invasive effectors in ACC cells. **A** Relative mRNA level and protein expression of metalloproteinase 8 (MMP-8) and representative immunoblot; **B** Relative mRNA level of metalloproteinase inhibitor 1 (*TIMP-1*); **C** Relative mRNA level of tumor metastasis suppressor 1 *KISS-1*, in H295R/TCF21 cells and respective controls. The experiments were performed in triplicate and repeated three times. The results were expressed as the mean ± SD. Statistical significance was assessed by unpaired *t* test

**Fig. 6:**
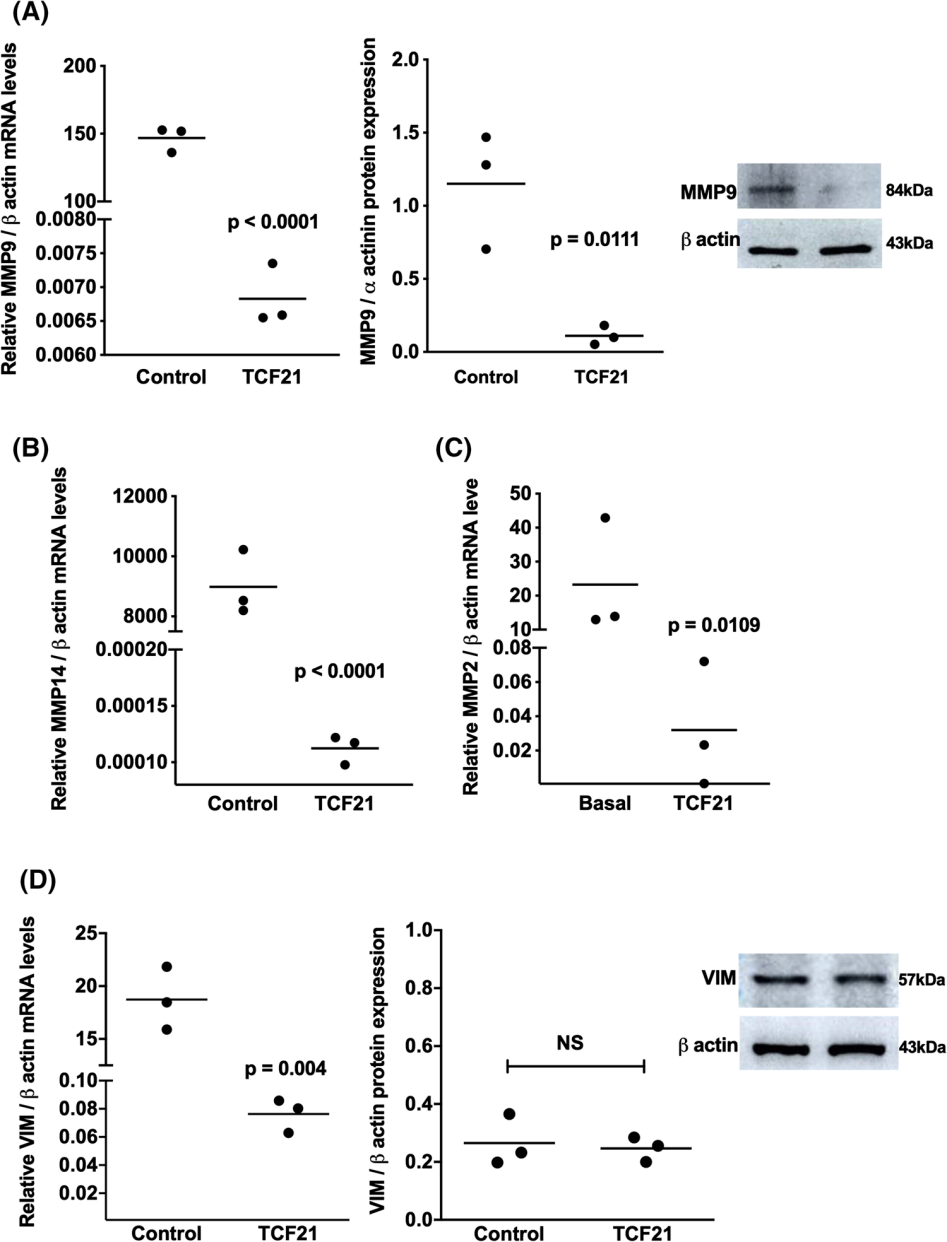
TCF21 downregulated the invasive effectors in ACC cells. **A** Relative mRNA level and protein expression of metalloproteinase 9 (MMP-9) and representative immunoblot; **B** Relative mRNA level of metalloproteinase 14 (*MMP-14*); **C** Relative mRNA level of metalloproteinase 2 (*MMP-2*), **D** Relative mRNA level and protein expression of *Vimentin* (VIM) and representative immunoblot in H295R/TCF21 cells and respective controls. The experiments were performed in triplicate and repeated three times. The results were expressed as the mean ± SD. Statistical significance was assessed by unpaired *t* test

**Fig. 7:**
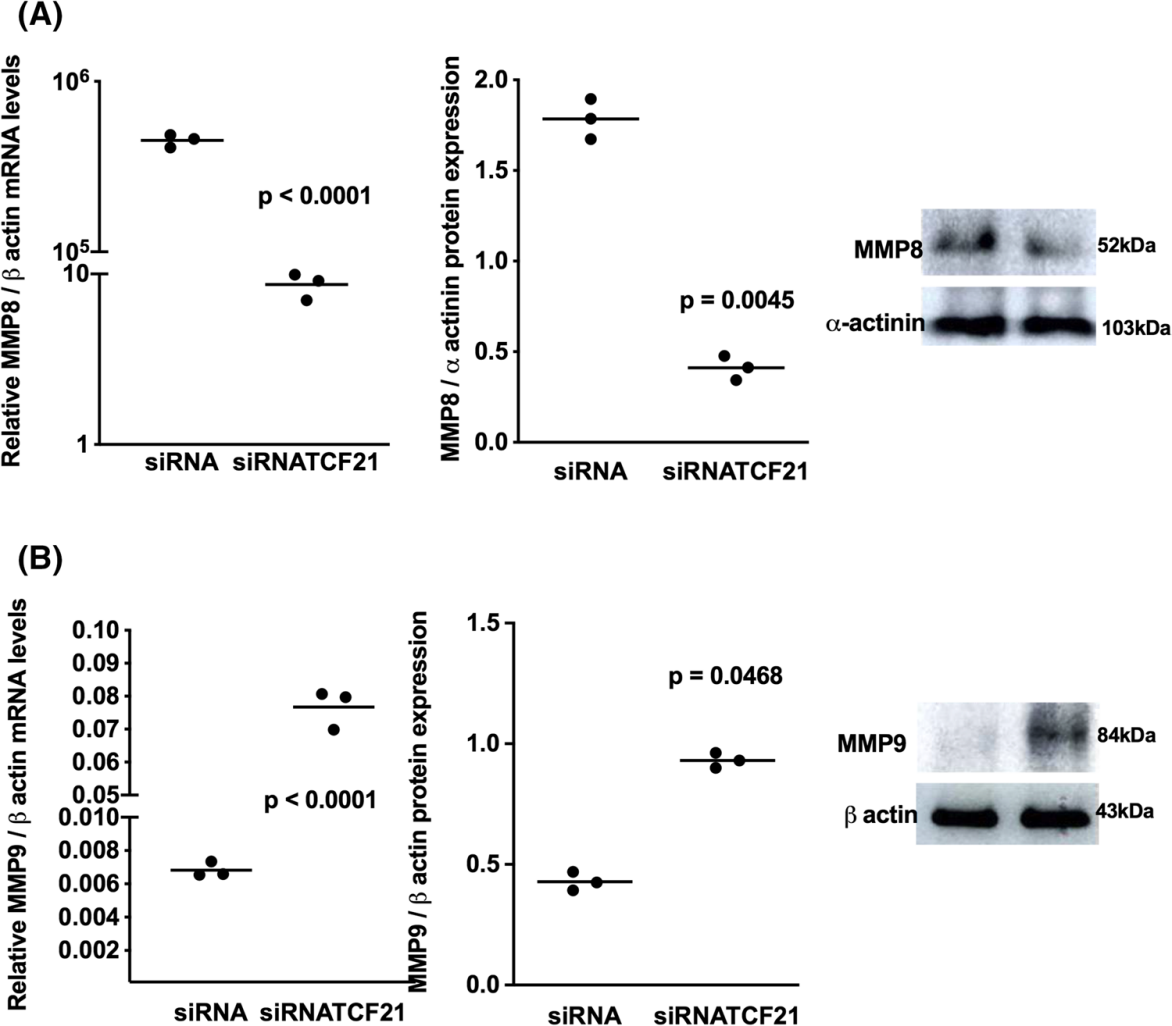
Inhibition of TCF21 inhibited MMP-8 and promote MMP-9 expression. **A** Relative mRNA level and protein expression of metalloproteinase 8 (MMP-8) and with representative immunoblot experiment; **B** Relative mRNA level and protein expression of metalloproteinase 9 (MMP-9) and representative immunoblot in ACAPed-T7siRNATCF21. The experiments were performed in triplicate and repeated three times. The results were expressed as the mean ± SD. Statistical significance was assessed by unpaired *t* test

To explore the potential molecular mechanism of TCF21 in cellular mobility of adrenocortical carcinoma cells, we analyzed the effect of TCF21 on the expression of metalloproteinases *MMP-2, MMP-8, MMP-9, MMP-14*, metalloproteinase inhibitor 1 precursor *TIMP-1, KISS-1* and *Vimentin* (VIM) in H295R/TCF21 cells and respective control. We observed significant increase in the mRNA level and protein expression of MMP-8, and mRNA level of *TIMP-1* and metastasis-suppressor *KISS* in H295R/TCF21 cells, in relation to control cells (Fig. 5A–C). In contrast, pro-invasive effectors, MMP-9 mRNA and protein, *MMP-14*, *MMP-2* and VIM mRNA were remarkably downregulated in H295R/TCF21 cells, although the decrease of VIM protein has not been observed (Fig. 6A–D). To confirm the involvement of MMP-8 and MMP-9 in the motility of ACTs, the mRNA and protein expression of both MMPs was evaluated when TCF21 was inhibited in ACAPed-T7siRNATCF21 cells (Fig. 7). Results showed that the inhibition of *TCF21* inhibited the mRNA level and protein expression of MMP-8 (Fig. 7A), in contrast to the increased of mRNA level and protein expression of MMP-9 (Fig. 7B).

### MMP-8 and MMP-9 are important factors for the motility of adrenocortical tumors

**Fig. 8:**
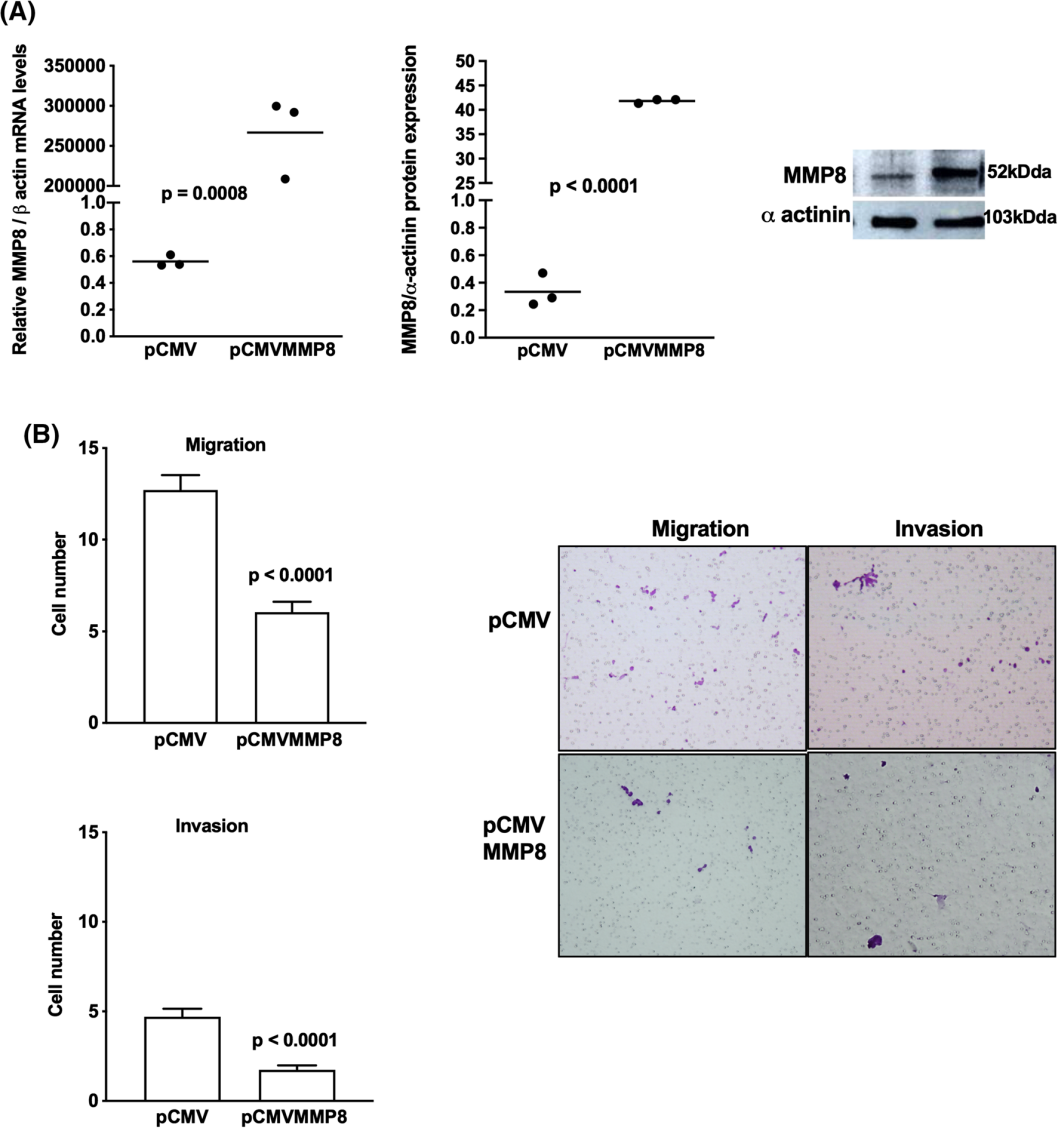
Overexpression of metalloproteinase MMP-8 in adrenocortical carcinoma cells. **A** MMP-8 expression in H295R cells transfected with pCMVMMP-8 and empty vector; representative immunoblot of MMP-8 expression; **B** migration and invasion assays in H295R transfected with pCMVMMP-8 and representative stained membranes. The experiments were performed in triplicate and repeated three times. Magnification of membranes (100×). The results were expressed as the mean ± SD. Statistical significance was assessed by unpaired *t* test

**Fig. 9:**
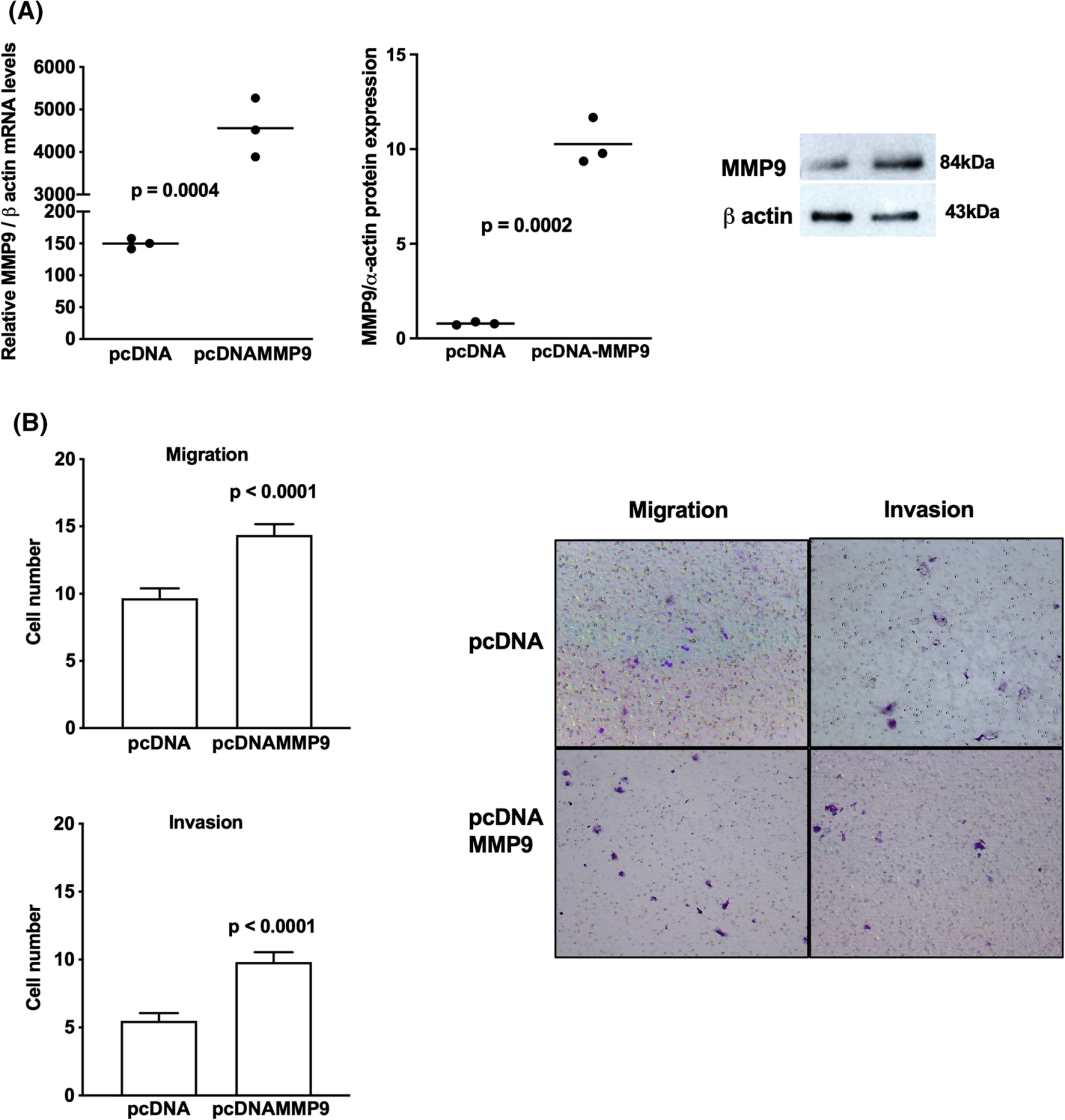
Overexpression of metalloproteinase MMP-9 in adrenocortical carcinoma cells. **A** MMP-9 expression in H295R cells transfected with pcDNAMMP-9 and empty vector; representative immunoblot of MMP-9 expression; **B** migration and invasion assays in H295R transfected with pcDNAMMP-9 and representative stained membranes. The experiments were performed in triplicate and repeated three times. Magnification of membranes (100×). The results were expressed as the mean ± SD. Statistical significance was assessed by unpaired *t* test

**Fig. 10:**
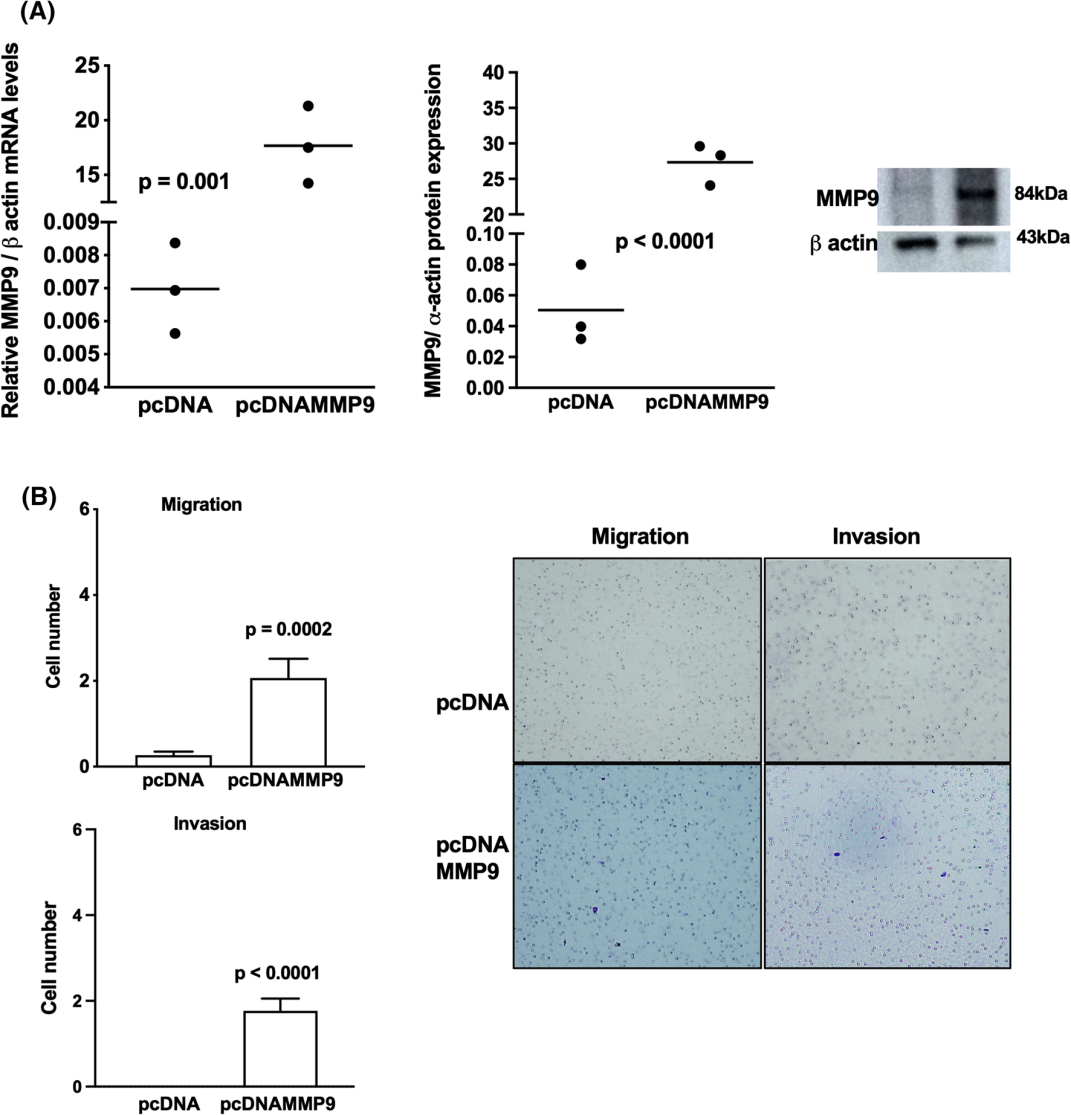
Overexpression of metalloproteinase in adrenocortical adenoma cells. **A** MMP-9 expression in ACAPed-T7 transfected with pcDNAMMP-9 and empty vector; representative immunoblot experiment of MMP-9; **B** migration and invasion assays in ACAPedT7 transfected with pcDNAMMP-9 and representative stained membranes. Magnification of membranes (100×). The experiments were performed in triplicate and repeated three times. The results were expressed as the mean ± SD. Statistical significance was assessed by unpaired *t* test

 To explore the effect of MMP-8 and MMP-9, both representatives of families of inhibitors and inducers of cell motility [26], in the migration and invasion of adrenocortical tumors, H295R cells were transfected with pCMVMMP-8 and pCMVMMM9 (Figs. 8 and 9). After MMP-8 and MMP-9 overexpression (Figs. 8A and 9A), MMP-8 decreased, and MMP-9 increased the migration and invasion capacity of H295R pCMVMMP-8 cells (Figs. 8B and 9B). The overexpression of MMP-9 in ACAPed-T7 cells (Fig. 10A) resulted in a gain in the motility of these cells (Fig. 10B). Together, we showed that MMP-8 and MMP-9 play an important role in adrenocortical tumor cell motility, which may suggest a role in the metastatic process.

## Discussion

In the present study, we demonstrated in functional studies that restoration of TCF21 expression in ACC cells results in reduction of migration and invasion in vitro. These results reinforce the tumor suppressor function of TCF21 in ACC, in accordance with our studies that demonstrated that TCF21 is markedly downregulated in adult ACCs compared with adenomas and normal tissue [12, 14]. The tumor suppressor function of TCF21 is also demonstrated in different tumors such as lung, colorectal and breast cancer, where the activation of TCF21 expression reduced cell growth, EMT and suppress migration and invasion [9, 27, 28]. In addition, downregulation of TCF21 has associated with promoter hypermethylation in different tumors [17, 18]. Here, we reported that TCF21 promoter hypermethylation is involved in the repressed expression of TCF21 observed in ACC cells, NCI-H295R and ACC-T36. Furthermore, the hypermethylation condition of TCF21 was reverted in H295R cell line with the 5-Aza treatment in a dose and time dependent manner. This result suggest that the promoter methylation is an important mechanism of epigenetic control of TCF21 expression in ACC, and it can be reverted at least in vitro. In other human tumors, hypermethylation is described as the mechanism preponderant for silencing TCF21 expression [9, 17, 29].

In order to study the biological function of TCF21 in H295R cells, we used transfection with pCMVMyc-Pod1 as an experimental method necessary to study the role of TCF21. The ability of migration and invasion were greatly inhibited in the TCF21 upregulated cells in comparison to negative control cells. In addition, and also important, the downregulation of TCF21 in an adrenocortical adenoma cell culture, enable the migration and invasive ability of tumor adrenocortical cells when compared to control cells. Together, these results suggest a role of TCF21 in the motility of ACC cells.

We further investigate the potential molecular mechanism regulated by TCF21 in adrenocortical tumor cells. The MMPs are a family of zinc-dependent endoproteases involved in tissue remodeling and degradation of various proteins in the extracellular matrix. Despite performing important extracellular actions several MMPs are known to function intracellularly in diverse tissue [30]. Alterations in MMP expression occur in normal biological processes such as cell proliferation, migration, and differentiation and have also been implicated in tumor progression and invasiveness. MMPs have been examined as potential therapeutic targets in various disorders as well as cancer [26]. In our study, the presence of TCF21 in ACC cells induced the increased of anti-invasive effectors, KISS-1, MMP-8 and TIMP-1 expression whereas the pro-invasive, MMP-9, MMP-14, MMP-2 and VIM were downregulated in ACC cells.

Arab and coworkers [29] found that TCF21 directly bind the promoter of KISS-1 gene, known as metastasis inhibition gene in a number of tissues, to enhance its expression in melanomas. In renal cancer, the expression of KISS-1 was downregulated with TCF21 gene silencing [31]. More recently, TCF21 was found to induce KISS-1 and reduce MMPs expression through the PI3K/Akt pathway in colorectal cancer [32]. In our study, KISS-1 was also upregulated significantly for TCF21 in ACC cells as well MMP-8 and TIMP-1. The anti-tumor properties of MMP-8 were first demonstrated in MMP-8-deficient mice [33]. The absence of MMP-8 strongly increases the incidence of skin tumors in these mice. Also, the overexpression of MMP-8 in metastatic breast cancer cells reduces their metastatic potential, and higher MMP-8 expression is correlated with the lower incidence of lymph node metastasis and good prognosis [34]. Thus, it was proposed that MMP-8 is a tumor protective factor, but as far as we know, there is no report of the action of TCF21 in regulating MMP-8 expression.

Tissue inhibitors of metalloproteinases (TIMPs) participate in controlling the local activities of MMPs in tissues. TIMPs are smaller, 22–30 kDa, and capable of binding and inactivating MMPs. Four TIMPs have been identified (TIMP-1 to TIMP4), that form noncovalent bonds with the latent and active forms of MMPs. Overexpression of TIMP-1, TIMP-2, and TIMP-3 reduces tumor growth [35]. Although TIMPs inhibit MMPS and have an antitumoral effect, they are also involved in the activation of MMPs, thus promoting tumor progression [36]. The TCF21 knockdown in visceral derived adipose stem cells was showed to suppress the expression of TIMP-1 in the remodeling of the extracellular matrix of adipose tissue [37]. Our data also showed downregulation of MMP pro-invasive MMPs, MMP-9, MMP-14 and MMP-2. MMP-9 or gelatinase B is a type IV collagenase produced by a variety of cells. It inhibits or stimulates the process of degradation of extracellular matrix during tissue remodeling which is essential for tumor invasion and metastasis. The MMP-9 enzyme is secreted as pro-MMP-9 which is an inactive form of MMP-9 and it is activated by various activators including MMP-2 and MMP-3 [38]. In addition, the MMP-9 and MMP-8 serum levels were evaluated in patients with adrenal tumors prior and after surgery. Increased MMP-9 and MMP-8 levels were noted in patients with ACA and ACC prior surgery. After surgery, MMP-8 and MMP-9 levels decreased significantly in patients with ACC whereas in patients with ACA the decreased was not statistically significant. However, no correlation between the levels of evaluated MMPs and tumor sizes were observed [39]. In other study, serum MMP-9 levels were used as diagnostic tool in determining the functioning status of benign adrenal tumors. The results suggested that MMP-9 may be useful in differentiating benign subclinical functioning adrenal tumors from benign nonfunctioning adrenal tumors [39]. TCF21 was overexpressed in SMMC-7721 hepatocellular carcinoma cell line and showed raised of KISS-1 and p53, and downregulated MMP-9 proteins and inhibition of migration ability [40].

The MMP-2 is a type IV collagenase or gelatinase A. Indeed, the MMP-2 is ubiquitous in many cells and tissues and is involved in different processes such as angiogenesis, tissue repair, and inflammation. The proMMP-2 is recruited to the cell surface and undergoes autocatalytic cleavage at the cell surface. The imbalance of MMP-2 and its inhibitors TIMP-1 contribute to tumor invasion and metastasis, and tumor progression [30]. The involvement of MMP-2 in cancer has been studied in different malignancies [29]. Investigation of 50 ACC and 50 ACA by immunohistochemical showed that MMP-2 was detected in 74% of ACC and 2% in ACA. In addition, strong MMP-2 expression was recognized as an unfavorable prognostic factor for ACC [41]. The MMP-2 inhibition by presence of TCF21 in ACC cells was also reported in colon rectal and ovarian cancer cells [32, 42], showing the TCF21 properties in decrease cell invasion of different types of tumors through inhibition of MMP-9 and MMP-2.

The MMP-14 was found to be highly expressed in different cancers. Thus, MMP-14 expression promotes migration, invasion and metastasis of tumor cells in vitro and in vivo [43]. The MMP-14 is a membrane MMP that its primary role is extracellular matrix (ECM) degradation, in fact, it is located at the leading edge of migrating cells. A critical catalytic domain was identified for the enhancement of cellular invasion by regulating cleavage of pro-MMP-2 to active MMP-2 [44]. Indeed, the MMP-14 are inhibited by TIMPs, RECK (GPI-anchored glycoprotein), chondroitin/heparan sulfate proteoglycans and the keratan sulfate Lumican [45]. Our data showed that MMP-14 was efficiently inhibited by TCF21 in ACC cells, by a mechanism not yet known but together with KISS-1, TIMP-1 and MMPs, MMP-14 inhibits the motility capacity of ACC cells.

In the present study, we found that the methylation is an important and reversible mechanism of epigenetic control of TCF21 expression in ACC, and that overexpression of TCF21 inhibits migration, and invasion of ACC cells. Furthermore, the expression of MMP-8, TIMP-1 and KISS were increased while MMP-9, MMP-14 and MMP-2 were decreased.

## Conclusion

In summary, our study revealed that restoration of TCF21 expression, that is epigenetically silenced in ACC cells, results in decreased migration and invasion. These results showed that tumor inhibitory functions of TCF21 may act promoting anti-invasive effectors like KISS-1, reverting the epithelial–mesenchymal transition and inhibiting the invasive ability of MMPs in ACC.

## Supplementary Information

Below is the link to the electronic supplementary material.

Additional file 1: Figure S1. TCF21 demethylation with 5-Aza-2′-deoxycytidine (5-Aza). A) Relative mRNA level of *TCF21* in H295R cells treated with different concentrations of 5-Aza 48 h; B) Relative mRNA level of *TCF21* in H295R cells treated with 100 μM 5-Aza in different times as indicated. The experiments were performed in triplicate and repeated three times. Statistical significance was assessed by One-way ANOVA and post-test from Tukey’s test; NS = not significant

Additional file 2: Table S1. Regulatory region of the TCF21 promoter
