# Supplementary figures and images for: Upregulation of TCF21 inhibits migration of adrenocortical carcinoma cells
Source: Discov Oncol. 2021 Jul 23;12:23. doi: 10.1007/s12672-021-00417-6 (PMC8777580; doi:10.1007/s12672-021-00417-6)

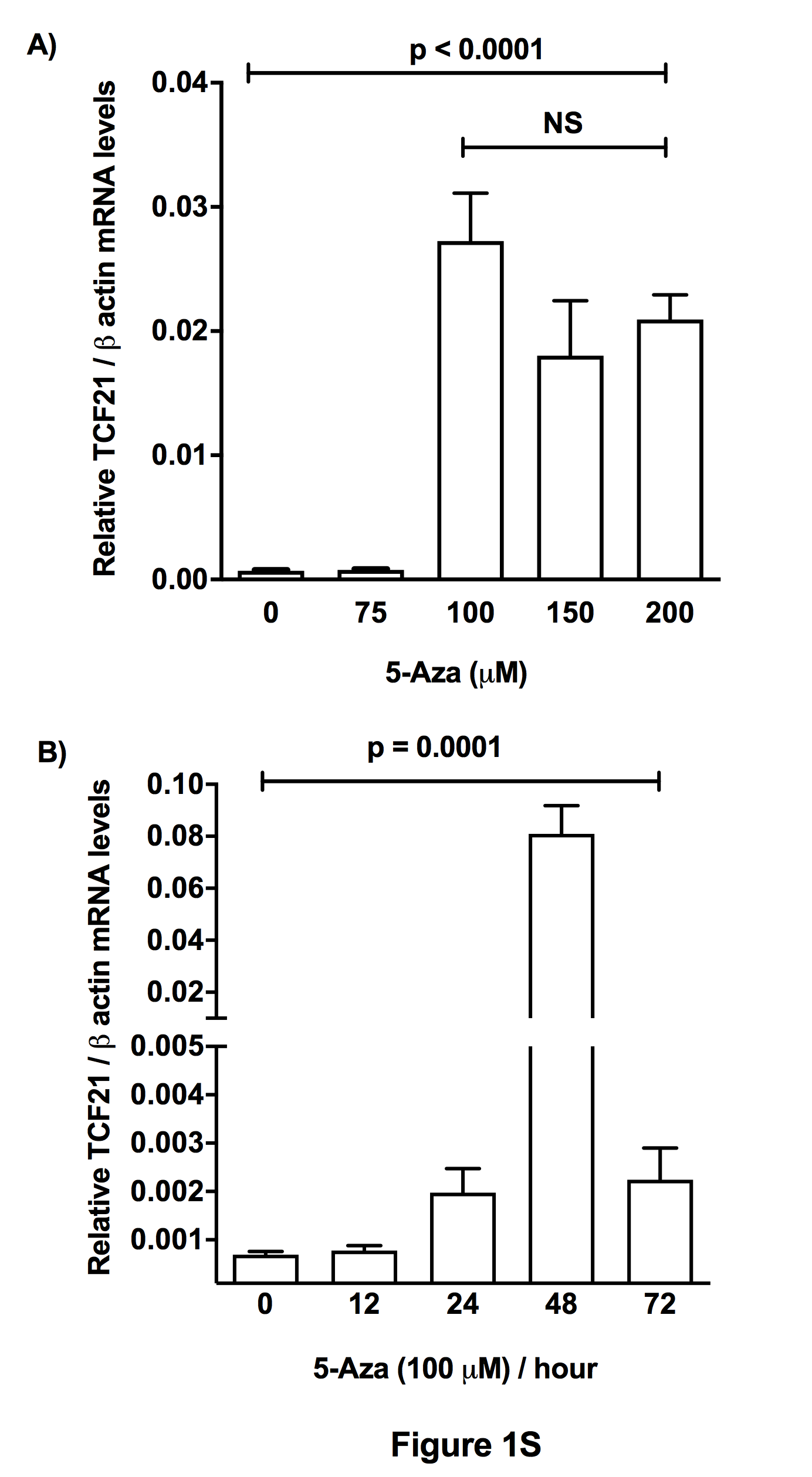

Supplement: Supplementary file 1 — Additional file 1: Figure S1. TCF21 demethylation with 5-Aza-2′-deoxycytidine (5-Aza). A) Relative mRNA level of TCF21 in H295R cells treated with different concentrations of 5-Aza 48 h; B) Relative mRNA level of TCF21 in H295R cells treated with 100 μM 5-Aza in different times as indicated. The experiments were performed in triplicate and repeated three times. Statistical significance was assessed by One-way ANOVA and post-test from Tukey’s test; NS = not significant [file 12672_2021_417_MOESM1_ESM.tiff]
