# Supplementary material for: Upregulation of TCF21 inhibits migration of adrenocortical carcinoma cells
Source: Discov Oncol. 2021 Jul 23;12:23. doi: 10.1007/s12672-021-00417-6 (PMC8777580; doi:10.1007/s12672-021-00417-6)
Supplement: Supplementary file 2 — Additional file 2: Table S1. Regulatory region of the TCF21 promoter [file 12672_2021_417_MOESM2_ESM.docx]

**Table 1S. Regulatory region of the TCF21 promoter**

(ENSG00000118526; r = 6: 133.889.335-133.895.553)

TTTTCCATCACCATAAAGATTCTAGGAAGCACTTGTCTTCATTTATCCCGGGAAGGTGGAAGATCTCGGTAAAGCCTTGGAGTCTTATGAGGTGTTGGCAGGATGCGGTTGATACTCCCTGTATTTTAATTCGTGTTTTCTTGGGAGGAGAGAGGGCATGGAGTGTAGGTGGAAGGCCCAGAAAGAGCCAAACGATCTAGTCGTTTTTCTAAAGTTCTCAGTAACAACCAGTTGTGACGTCTTAAGCACTTGTCTTCATTTATCCCGAGAGAGAAAGAAGGCCTTTTACAGATGGACAGAACATGCTGCTTCTCGGCCCCGGCCACAGTTGGGAGAAGGTGGCTGGTAAGTACATGATGCAGTTGAGTTGATTTACATTACAAGTTGCAAATCAGGGTCTGTGCATGAGTTTCC GTAAGTACATGATGCAGTTGAGTTGATTTACATTACAAGTTGCAAATCAGGGTCTGTGCATGAGTTTCCCTCCGGTTGTGAAAGGGGTCTAAGCGCTTTGCTGGGGCCTGGCAGGCCCCTCCCTGGGCTGCAAGGATCCGCCCCTCTATTCCCCAGATAAATTCCTAGTGTCCACCAAATTCCTCAGCGCTCGCTCACCCTCCTCTACGG
